# Poly[[di­aqua­deca-μ_2_-cyanido-κ^20^ *C*:*N*-hexa­cyanido-κ^6^ *C*-bis­(μ_2_-5-methyl­pyrimidine-κ^2^ *N*:*N*′)bis­(5-methyl­pyrimidine-κ*N*)tricopper(II)ditungstate(V)] dihydrate]

**DOI:** 10.1107/S1600536814000166

**Published:** 2014-01-18

**Authors:** Yoshihide Tsunobuchi, Souhei Kaneko, Koji Nakabayashi, Shin-ichi Ohkoshi

**Affiliations:** a Department of Chemistry, School of Science, University of Tokyo, 7-3-1 Hongo, Bunkyo-Ku, Tokyo 113-0033, Japan

## Abstract

In the title complex, {[Cu_3_[W(CN)_8_]_2_(C_5_H_6_N_2_)_4_(H_2_O)_2_]·2H_2_O}_*n*_, the coordination polyhedron of the eight-coordinated W^V^ atom is a bicapped trigonal prism, in which five CN groups are bridged to Cu^II^ ions, and the other three CN groups are terminally bound. Two of the Cu^II^ ions lie on a centre of inversion and each of the three independent Cu^II^ cations is pseudo-octahedrally coordinated. In the crystal structure, cyanido-bridged-Cu—W—Cu layers are linked by pillars involving the third independent Cu^II^ ion, generating a three-dimensional network with non-coordinating water mol­ecules and 5-methyl­pyrimidine mol­ecules. O—H⋯O and O—H⋯N hydrogen bonds involve the coordinating and non-coordin­ating water mol­ecules, the CN groups and the 5-methyl­pyrimidine mol­ecules.

## Related literature   

For background to functional three-dimensional networks, see: Catala *et al.* (2005 ▶); Garde *et al.* (1999 ▶); Herrera *et al.* (2004 ▶, 2008 ▶); Imoto *et al.* (2012 ▶); Leipoldt *et al.* (1994 ▶); Ohkoshi & Tokoro (2012 ▶); Ohkoshi *et al.* (2011 ▶); Sieklucka *et al.* (2009 ▶); Zhong *et al.* (2000 ▶). For related structures, see: Ohkoshi *et al.* (2007 ▶, 2012 ▶); Podgajny *et al.* (2002 ▶).
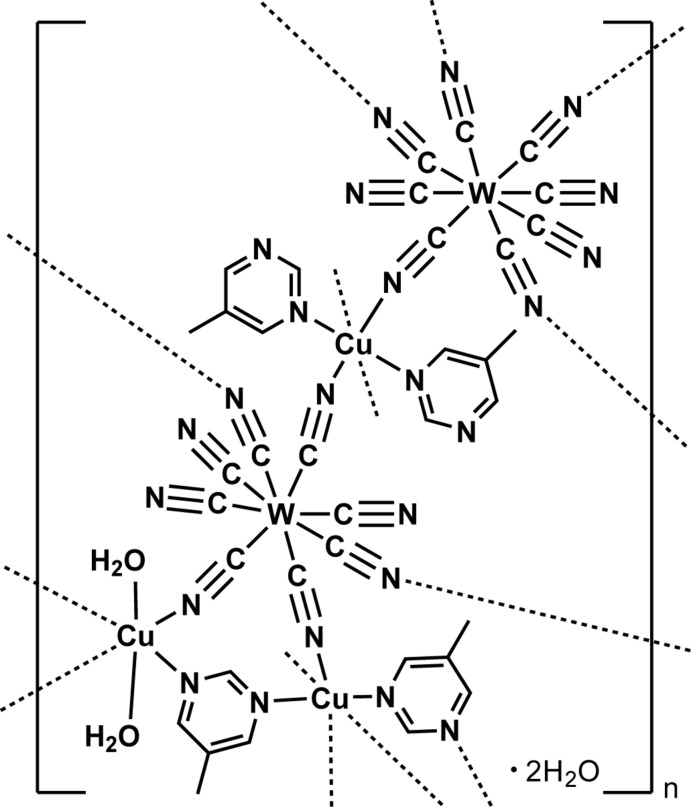


## Experimental   

### 

#### Crystal data  

[Cu_3_W_2_(CN)_16_(C_5_H_6_N_2_)_4_(H_2_O)_2_]·2H_2_O
*M* *_r_* = 1423.19
Triclinic, 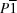

*a* = 7.5953 (4) Å
*b* = 11.8232 (7) Å
*c* = 14.7017 (8) Å
α = 79.614 (1)°
β = 84.824 (2)°
γ = 73.090 (1)°
*V* = 1241.45 (12) Å^3^
*Z* = 1
Mo *K*α radiation
μ = 5.94 mm^−1^
*T* = 296 K
0.16 × 0.10 × 0.05 mm

#### Data collection  

Rigaku R-AXIS RAPID diffractometer
Absorption correction: multi-scan (*ABSCOR*; Higashi, 1995 ▶) *T*_min_ = 0.452, *T*_max_ = 0.772
12240 measured reflections
5666 independent reflections
5465 reflections with *I* > 2σ(*I*)
*R*_int_ = 0.033

#### Refinement  

*R*[*F* ^2^ > 2σ(*F* ^2^)] = 0.029
*wR*(*F* ^2^) = 0.079
*S* = 1.24
5666 reflections
333 parameters
6 restraints
H atoms treated by a mixture of independent and constrained refinement
Δρ_max_ = 3.02 e Å^−3^
Δρ_min_ = −0.86 e Å^−3^

### 

Data collection: *PROCESS-AUTO* (Rigaku, 1998 ▶); cell refinement: *PROCESS-AUTO*; data reduction: *CrystalStructure* (Rigaku, 2007 ▶); program(s) used to solve structure: *SHELXS97* (Sheldrick, 2008 ▶); program(s) used to refine structure: *SHELXL97* (Sheldrick, 2008 ▶); molecular graphics: *PyMOLWin* (DeLano, 2007 ▶); software used to prepare material for publication: *publCIF* (Westrip, 2010 ▶).

## Supplementary Material

Crystal structure: contains datablock(s) I, shelxl. DOI: 10.1107/S1600536814000166/tk5281sup1.cif

Structure factors: contains datablock(s) I. DOI: 10.1107/S1600536814000166/tk5281Isup2.hkl

CCDC reference:

Additional supporting information:  crystallographic information; 3D view; checkCIF report

## Figures and Tables

**Table 1:** Hydrogen-bond geometry (Å, °)

*D*—H⋯*A*	*D*—H	H⋯*A*	*D*⋯*A*	*D*—H⋯*A*
O1—H1⋯N6^i^	0.92 (2)	1.86 (2)	2.771 (5)	167 (5)
O1—H2⋯O2	0.93 (2)	1.79 (2)	2.700 (4)	165 (4)
O2—H3⋯N12^ii^	0.95 (2)	2.00 (3)	2.914 (5)	161 (4)
O2—H4⋯N2^iii^	0.93 (2)	2.02 (2)	2.944 (5)	169 (6)

Symmetry codes: (i) 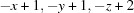; (ii) 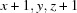; (iii) 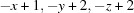.

**Fig. 1.**
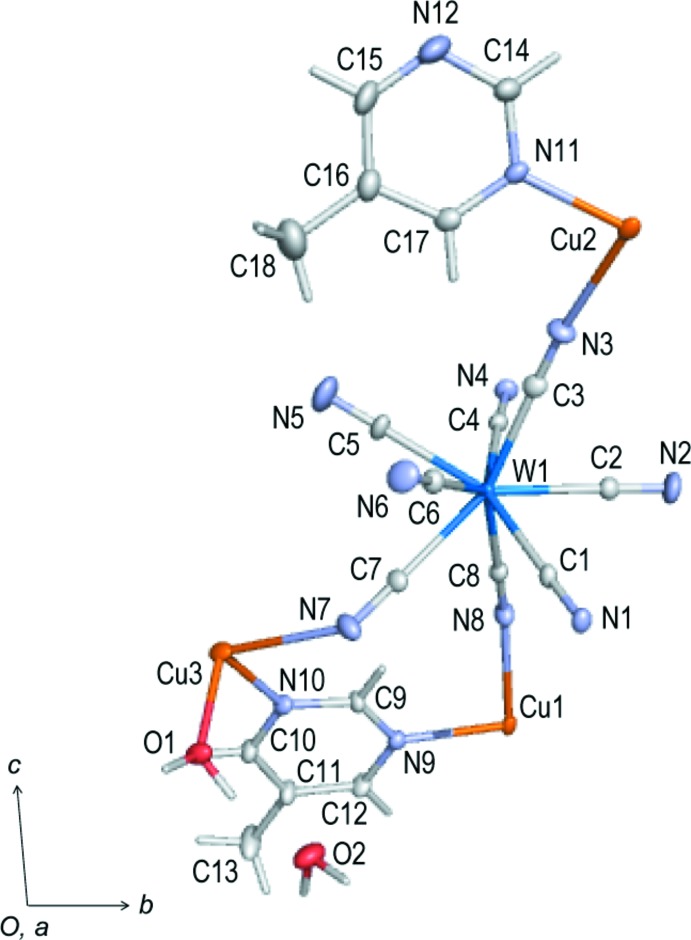
Displacement ellipsoid plot (30% probability level) of the atoms comprising the asymmetric unit of {[Cu3[W(CN)8]2(C5H6N2)4(H2O)2]·2H2O}n. Symmetry codes: (i) +x,+y,+z and (ii) -x,-y,-z.

**Fig. 2.**
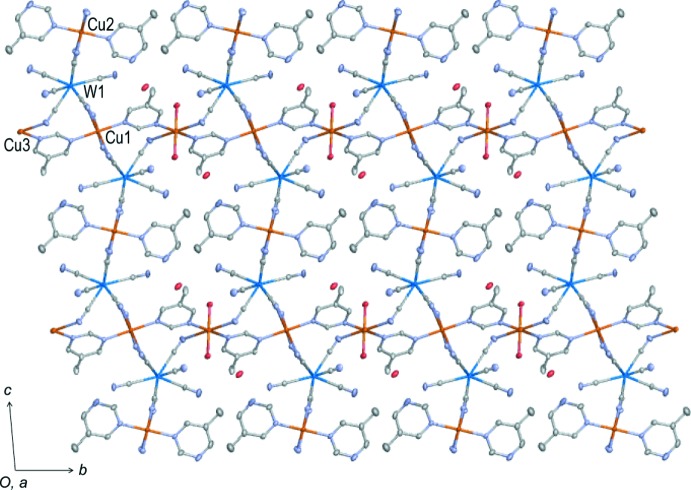
Crystal structure of {[Cu3[W(CN)8]2(C5H6N2)4(H2O)2]·2H2O}n along the a axis. Blue, orange, gray, light blue, and red represent W, Cu, C, N, and O atoms, respectively. Hydrogen atoms are omitted for clarity.

**Fig. 3.**
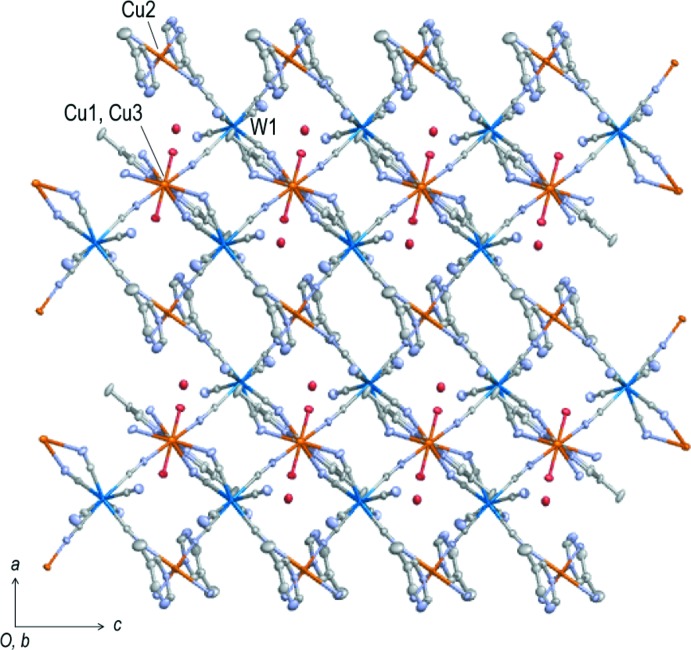
Crystal structure of {[Cu3[W(CN)8]2(C5H6N2)4(H2O)2]·2H2O}n along the b axis. Blue, orange, gray, light blue, and red represent W, Cu, C, N, and O atoms, respectively. Hydrogen atoms are omitted for clarity.

## supplementary crystallographic information

### 2.1. Synthesis and crystallization

The title compound was prepared by reacting an aqueous solution of Cs_3_[W(CN)_8_]·2H_2_O (1.2 × 10 ^-2^ mol dm^-3^) with a mixed aqueous solution of CuCl_2_·2H_2_O (1.8 × 10 ^-2^ mol dm^-3^), 5-methyl­pyrimidine (2.4 × 10 ^-2^ mol dm^-3^) at room temperature. The prepared compound was a green plate-type crystal. Elemental analyses: calcd for Cu_3_[W(CN)_8_]_2_(5-methyl­pyrimidine)_4_·4H_2_O, Calculated: Cu, 13.40; W, 25.83; C, 30.38; H, 2.27; N, 23.63%. Found: Cu, 13.12; W, 25.96; C, 30.05; H, 2.35; N, 23.64%. In the Infrared (IR) spectra, cyano stretching peaks were observed at 2204, 2194, 2169, 2161, 2148, and 2142 cm^-1^.

### 2.2. Refinement

The H atoms of the 5-methyl­pyrimidine molecules were placed in calculated positions with C—H = 0.95 Å, and refined using a riding model with *U*_iso_(H) = 1.2 *U*_eq_(C). The H atoms of water molecules were placed by using restraints of 0.96 (2) Å for O—H distances and DANG of 1.5 (4) Å for H—H distances. The maximum and minimum residual electron density peaks were located 0.74 and 1.60 Å, respectively, from the W1 and C3 atoms.

### 3. Results and discussion

Synthesis of various kinds of three-dimensional network complexes exhibiting long-range magnetic ordering is an important issue. From this perspective, o­cta­cyano­metalate [*M*(CN)_8_] (*M* = Mo, W, Nb)-based magnets have been studied because they show high *T*_C_ (Garde *et al.*, 1999; Zhong *et al.*, 2000; Herrera *et al.*, 2008; Sieklucka *et al.*, 2009; Imoto *et al.*, 2012) and functionalities such as photomagnetism (Herrera *et al.*, 2004; Catala *et al.*, 2005; Ohkoshi *et al.*, 2011, 2012) and chemically sensitive magnetism (Ohkoshi *et al.*, 2007). In addition, o­cta­cyano­metalates have an advantage to construct various crystal structures due to the versatility that they can adopt different spatial configurations depending on their chemical environment, *e.g.*, square anti­prism (*D*_4d_), dodecahedron (*D*_2d_), and bicapped trigonal prism (*C*_2v_) (Leipoldt *et al.*, 1994). Several o­cta­cyano­metalate-based magnets of Cu—W systems such as {[Cu_3_[W(CN)_8_]_2_]·3.4H_2_O}_n_ (Garde *et al.*, 1999), {[Cu_3_[W(CN)_8_]_2_(pyrimidine)_2_]·8H_2_O}_n_ (Ohkoshi *et al.*, 2007), {[Cu_3_[W(CN)_8_]_2_(pyrimidine)_4_]·4H_2_O}_n_ (Ohkoshi *et al.*, 2012), {[(tetrenH_5_)_0.8_Cu_4_[W(CN)_8_]_4_]·7.2H_2_O}_n_ (Podgajny *et al.*, 2002), have been reported. Here, we present a new candidate for copper-o­cta­cyano­tungstate-based magnets, {[Cu_3_[W(CN)_8_]_2_(5-methyl­pyrimidine)_4_(H_2_O)_2_]·2H_2_O}_n_.

The asymmetric unit of the present compound consists of a [W(CN)_8_]^3-^ anion, a one-half of [Cu1(5-methyl­pyrimidine)_2_]^2+^ cation, a one-half of [Cu2(5-methyl­pyrimidine)_2_]^2+^ cation, a one-half of [Cu3(H_2_O)_2_]^2+^ cation, and a water molecule (Fig. 1). The coordination geometry of W is an eight-coordinated bicapped trigonal prism, where five CN groups of [W(CN)_8_] are bridged to Cu^2+^ ions (two Cu1, two Cu2 and one Cu3), and the other three CN groups are free. The coordination geometries of the three types of Cu^2+^ ions (Cu1, Cu2 and Cu3) are six-coordinated pseudo-o­cta­hedron. Cu1 is coordinated to four nitro­gen atoms of CN ligands, two nitro­gen atoms of 5-methyl­pyrimidine molecules. Cu2 is coordinated to four nitro­gen atoms of CN ligands, two nitro­gen atoms of 5-methyl­pyrimidine molecules. Cu3 is coordinated to two nitro­gen atoms of CN ligands, two nitro­gen atoms of 5-methyl­pyrimidine molecules, and two oxygen atoms of H_2_O molecules. The cyano-bridged-Cu1—W—Cu2 layers are linked by Cu3 pillar unit (Figs. 2 and 3) and then, involving non-coordinated water molecules, the 3-D structure is constructed. In the crystal structure, the coordinated water make hydrogen bonds with the non-coordinated water (O1—H2···O2, 2.700 (4)) and the CN groups (O1—H1···N6, 2.771 (5)). Besides, hydrogen bonds between the non-coordinated water and the CN groups (O2—H4···N2, 2.944 (5)) or the 5-methyl­pyrimidine molecules (O2—H3···N12, 2.914 (5)).

The magnetization *versus*. temperature curve at 10 Oe showed a spontaneous magnetization with a Curie temperature (*T*_C_) of 10 K, the coercive field (*H*_c_) of 150 Oe at 2 K, and, the saturation magnetization (*M*_s_) value of 3.1 µ_B_. This *M*_s_ value agrees with the expected value of 3.0 µ_B_, indicating that this compound is a ferrimagnet in which W^V^ (*S* = 1/2) and Cu^II^ (*S* = 1/2, Cu1 and Cu2) in the layer are ferromagnetically coupled and W^V^ and the bridged Cu^II^ (*S* = 1/2, Cu3) are anti­ferromagnetically coupled.

### Crystal data

**Table:**

[Cu_3_W_2_(CN)_16_(C_5_H_6_N_2_)_4_(H_2_O)_2_]·2H_2_O	*Z* = 1
*M**_r_* = 1423.19	*F*(000) = 683
Triclinic, *P*1	*D*_x_ = 1.904 Mg m^−^^3^
Hall symbol: -P 1	Mo *K*α radiation, λ = 0.71075 Å
*a* = 7.5953 (4) Å	Cell parameters from 10947 reflections
*b* = 11.8232 (7) Å	θ = 3.0–27.5°
*c* = 14.7017 (8) Å	µ = 5.94 mm^−^^1^
α = 79.614 (1)°	*T* = 296 K
β = 84.824 (2)°	Platelet, green
γ = 73.090 (1)°	0.16 × 0.10 × 0.05 mm
*V* = 1241.45 (12) Å^3^	

### Data collection

**Table:**

Rigaku R-AXIS RAPID diffractometer	5666 independent reflections
Radiation source: fine-focus sealed tube	5465 reflections with *I* > 2σ(*I*)
Graphite monochromator	*R*_int_ = 0.033
Detector resolution: 10.00 pixels mm^-1^	θ_max_ = 27.5°, θ_min_ = 3.0°
ω scans	*h* = −9→9
Absorption correction: multi-scan (*ABSCOR*; Higashi, 1995)	*k* = −15→14
*T*_min_ = 0.452, *T*_max_ = 0.772	*l* = −19→19
12240 measured reflections	

### Refinement

**Table:**

Refinement on *F*^2^	Primary atom site location: structure-invariant direct methods
Least-squares matrix: full	Secondary atom site location: difference Fourier map
*R*[*F*^2^ > 2σ(*F*^2^)] = 0.029	Hydrogen site location: inferred from neighbouring sites
*wR*(*F*^2^) = 0.079	H atoms treated by a mixture of independent and constrained refinement
*S* = 1.24	*w* = 1/[σ^2^(*F*_o_^2^) + (0.0422*P*)^2^ + 0.3667*P*] where *P* = (*F*_o_^2^ + 2*F*_c_^2^)/3
5666 reflections	(Δ/σ)_max_ = 0.003
333 parameters	Δρ_max_ = 3.02 e Å^−^^3^
6 restraints	Δρ_min_ = −0.86 e Å^−^^3^

### Special details

**Table:**

Geometry. All e.s.d.'s (except the e.s.d. in the dihedral angle between two l.s. planes) are estimated using the full covariance matrix. The cell e.s.d.'s are taken into account individually in the estimation of e.s.d.'s in distances, angles and torsion angles; correlations between e.s.d.'s in cell parameters are only used when they are defined by crystal symmetry. An approximate (isotropic) treatment of cell e.s.d.'s is used for estimating e.s.d.'s involving l.s. planes.
Refinement. Refinement of *F*^2^ against ALL reflections. The weighted *R*-factor *wR* and goodness of fit *S* are based on *F*^2^, conventional *R*-factors *R* are based on *F*, with *F* set to zero for negative *F*^2^. The threshold expression of *F*^2^ > σ(*F*^2^) is used only for calculating *R*-factors(gt) *etc*. and is not relevant to the choice of reflections for refinement. *R*-factors based on *F*^2^ are statistically about twice as large as those based on *F*, and *R*- factors based on ALL data will be even larger.

### Fractional atomic coordinates and isotropic or equivalent isotropic displacement parameters (Å^2^)

**Table:**

	*x*	*y*	*z*	*U*_iso_*/*U*_eq_	
W1	0.548569 (15)	0.875339 (10)	0.768726 (8)	0.01497 (6)	
Cu1	1.0000	1.0000	1.0000	0.01979 (14)	
Cu2	0.0000	1.0000	0.5000	0.02293 (14)	
Cu3	1.0000	0.5000	1.0000	0.02754 (15)	
O1	0.9257 (4)	0.4659 (3)	1.12961 (19)	0.0325 (6)	
O2	0.8939 (5)	0.6502 (3)	1.2207 (3)	0.0446 (8)	
N1	0.2221 (4)	0.9864 (3)	0.9186 (2)	0.0253 (6)	
N2	0.4282 (6)	1.1699 (3)	0.7155 (3)	0.0439 (10)	
N3	0.2648 (5)	0.9438 (3)	0.5964 (3)	0.0352 (8)	
N4	0.8430 (4)	0.9479 (3)	0.6057 (2)	0.0274 (7)	
N5	0.7737 (7)	0.6301 (4)	0.6849 (4)	0.0615 (13)	
N6	0.2689 (5)	0.7008 (4)	0.8104 (3)	0.0437 (9)	
N7	0.6975 (5)	0.6771 (3)	0.9555 (3)	0.0376 (9)	
N8	0.8384 (5)	0.9585 (3)	0.8789 (2)	0.0309 (7)	
N9	1.1037 (4)	0.8172 (3)	1.0463 (2)	0.0222 (6)	
N10	1.1143 (4)	0.6182 (3)	1.0366 (2)	0.0240 (6)	
N11	0.0743 (4)	0.8306 (3)	0.4682 (2)	0.0271 (7)	
N12	0.0945 (6)	0.6994 (4)	0.3619 (3)	0.0550 (12)	
C1	0.3400 (5)	0.9497 (3)	0.8680 (2)	0.0219 (7)	
C2	0.4714 (5)	1.0702 (4)	0.7326 (3)	0.0262 (8)	
C3	0.3692 (5)	0.9207 (4)	0.6532 (3)	0.0272 (8)	
C4	0.7413 (5)	0.9202 (3)	0.6609 (2)	0.0219 (7)	
C5	0.6973 (6)	0.7146 (3)	0.7133 (3)	0.0323 (9)	
C6	0.3645 (5)	0.7610 (3)	0.7957 (3)	0.0269 (8)	
C7	0.6405 (5)	0.7462 (3)	0.8920 (3)	0.0260 (8)	
C8	0.7391 (5)	0.9303 (3)	0.8395 (3)	0.0231 (7)	
C9	1.0448 (5)	0.7368 (3)	1.0154 (3)	0.0227 (7)	
H9	0.9472	0.7651	0.9758	0.027*	
C10	1.2529 (5)	0.5779 (3)	1.0949 (3)	0.0281 (8)	
H10	1.3036	0.4958	1.1111	0.034*	
C11	1.3227 (5)	0.6550 (3)	1.1315 (3)	0.0286 (8)	
C12	1.2427 (5)	0.7751 (4)	1.1046 (3)	0.0292 (8)	
H12	1.2865	0.8296	1.1276	0.035*	
C13	1.4754 (7)	0.6078 (4)	1.1980 (4)	0.0532 (14)	
H13A	1.5110	0.5219	1.2077	0.064*	
H13B	1.4340	0.6354	1.2558	0.064*	
H13C	1.5790	0.6360	1.1730	0.064*	
C14	0.0516 (6)	0.8084 (4)	0.3854 (3)	0.0413 (10)	
H14	0.0022	0.8735	0.3403	0.050*	
C15	0.1642 (7)	0.6077 (4)	0.4271 (4)	0.0501 (12)	
H15	0.1950	0.5309	0.4125	0.060*	
C16	0.1936 (7)	0.6203 (4)	0.5156 (4)	0.0423 (11)	
C17	0.1426 (6)	0.7371 (4)	0.5327 (3)	0.0332 (9)	
H17	0.1568	0.7507	0.5916	0.040*	
C18	0.2772 (12)	0.5161 (6)	0.5890 (5)	0.083 (2)	
H18A	0.2844	0.5452	0.6451	0.099*	
H18B	0.3987	0.4749	0.5684	0.099*	
H18C	0.2019	0.4620	0.6003	0.099*	
H1	0.868 (7)	0.410 (3)	1.159 (3)	0.052 (15)*	
H2	0.913 (6)	0.520 (3)	1.170 (3)	0.047 (14)*	
H3	0.981 (5)	0.653 (5)	1.262 (3)	0.044 (14)*	
H4	0.785 (4)	0.699 (5)	1.244 (4)	0.08 (2)*	

### Atomic displacement parameters (Å^2^)

**Table:**

	*U*^11^	*U*^22^	*U*^33^	*U*^12^	*U*^13^	*U*^23^
W1	0.01514 (9)	0.01469 (9)	0.01571 (9)	−0.00529 (6)	0.00316 (6)	−0.00411 (6)
Cu1	0.0179 (3)	0.0139 (3)	0.0246 (3)	−0.0026 (2)	0.0084 (2)	−0.0033 (2)
Cu2	0.0273 (3)	0.0226 (3)	0.0205 (3)	−0.0112 (3)	0.0107 (3)	−0.0063 (3)
Cu3	0.0403 (4)	0.0269 (3)	0.0249 (3)	−0.0228 (3)	0.0015 (3)	−0.0074 (3)
O1	0.0436 (16)	0.0348 (16)	0.0273 (14)	−0.0244 (14)	0.0064 (12)	−0.0076 (12)
O2	0.0377 (17)	0.056 (2)	0.0461 (19)	−0.0118 (16)	0.0010 (15)	−0.0275 (17)
N1	0.0222 (14)	0.0207 (15)	0.0297 (16)	−0.0032 (12)	0.0065 (13)	−0.0039 (13)
N2	0.052 (2)	0.0182 (18)	0.057 (3)	−0.0024 (17)	−0.003 (2)	−0.0084 (17)
N3	0.0321 (18)	0.042 (2)	0.0337 (19)	−0.0125 (16)	−0.0125 (15)	−0.0027 (16)
N4	0.0259 (15)	0.0313 (17)	0.0242 (15)	−0.0099 (14)	0.0073 (13)	−0.0038 (13)
N5	0.076 (3)	0.035 (2)	0.068 (3)	−0.006 (2)	0.019 (3)	−0.024 (2)
N6	0.048 (2)	0.043 (2)	0.052 (2)	−0.0315 (19)	0.0051 (18)	−0.0095 (18)
N7	0.050 (2)	0.0295 (19)	0.0361 (19)	−0.0172 (17)	−0.0145 (17)	0.0056 (16)
N8	0.0343 (17)	0.0225 (16)	0.0375 (18)	−0.0093 (14)	−0.0094 (15)	−0.0026 (14)
N9	0.0204 (14)	0.0214 (15)	0.0237 (15)	−0.0041 (12)	0.0020 (12)	−0.0058 (13)
N10	0.0310 (16)	0.0206 (15)	0.0244 (15)	−0.0138 (13)	0.0000 (13)	−0.0033 (12)
N11	0.0303 (16)	0.0279 (16)	0.0260 (16)	−0.0092 (14)	0.0038 (13)	−0.0122 (14)
N12	0.064 (3)	0.051 (3)	0.053 (3)	−0.003 (2)	−0.012 (2)	−0.031 (2)
C1	0.0194 (16)	0.0170 (16)	0.0269 (17)	−0.0041 (13)	0.0024 (14)	−0.0012 (14)
C2	0.0224 (18)	0.026 (2)	0.0273 (19)	−0.0029 (15)	0.0001 (15)	−0.0046 (16)
C3	0.0276 (18)	0.0292 (19)	0.0279 (18)	−0.0138 (16)	0.0020 (16)	−0.0045 (16)
C4	0.0210 (16)	0.0225 (17)	0.0212 (17)	−0.0045 (14)	0.0014 (14)	−0.0051 (14)
C5	0.040 (2)	0.0203 (18)	0.034 (2)	−0.0060 (17)	0.0104 (18)	−0.0099 (16)
C6	0.0270 (18)	0.0239 (18)	0.0299 (19)	−0.0087 (15)	0.0015 (15)	−0.0032 (15)
C7	0.0311 (19)	0.0220 (18)	0.0274 (19)	−0.0125 (16)	−0.0013 (16)	−0.0020 (16)
C8	0.0279 (17)	0.0190 (16)	0.0233 (17)	−0.0085 (14)	0.0001 (15)	−0.0030 (14)
C9	0.0266 (18)	0.0149 (16)	0.0270 (18)	−0.0075 (14)	−0.0051 (15)	0.0008 (14)
C10	0.0301 (18)	0.0183 (17)	0.037 (2)	−0.0066 (15)	−0.0039 (16)	−0.0065 (15)
C11	0.0229 (17)	0.0195 (17)	0.042 (2)	−0.0009 (15)	−0.0094 (16)	−0.0066 (16)
C12	0.0270 (18)	0.0244 (19)	0.041 (2)	−0.0109 (15)	−0.0042 (17)	−0.0110 (17)
C13	0.047 (3)	0.030 (2)	0.085 (4)	−0.001 (2)	−0.038 (3)	−0.014 (3)
C14	0.049 (3)	0.040 (2)	0.032 (2)	0.000 (2)	−0.008 (2)	−0.0137 (19)
C15	0.054 (3)	0.030 (2)	0.065 (3)	0.002 (2)	−0.006 (3)	−0.023 (2)
C16	0.047 (3)	0.027 (2)	0.050 (3)	−0.004 (2)	−0.001 (2)	−0.009 (2)
C17	0.042 (2)	0.033 (2)	0.028 (2)	−0.0157 (19)	0.0000 (18)	−0.0070 (18)
C18	0.120 (6)	0.043 (3)	0.070 (4)	−0.007 (4)	−0.010 (4)	0.006 (3)

### Geometric parameters (Å, º)

**Table:**

W1—C1	2.156 (3)	N4—C4	1.141 (5)
W1—C8	2.160 (4)	N4—Cu2^ii^	1.991 (3)
W1—C4	2.160 (4)	N5—C5	1.131 (5)
W1—C5	2.167 (4)	N6—C6	1.139 (5)
W1—C3	2.167 (4)	N7—C7	1.148 (5)
W1—C7	2.171 (4)	N8—C8	1.144 (5)
W1—C6	2.178 (4)	N9—C9	1.323 (5)
W1—C2	2.182 (4)	N9—C12	1.341 (5)
Cu1—N1^i^	1.962 (3)	N10—C9	1.335 (5)
Cu1—N1^ii^	1.962 (3)	N10—C10	1.338 (5)
Cu1—N9	2.081 (3)	N11—C14	1.328 (5)
Cu1—N9^iii^	2.081 (3)	N11—C17	1.329 (6)
Cu1—N8^iii^	2.444 (3)	N12—C15	1.326 (7)
Cu1—N8	2.444 (3)	N12—C14	1.334 (6)
Cu2—N4^iv^	1.991 (3)	C9—H9	0.9300
Cu2—N4^v^	1.991 (3)	C10—C11	1.383 (5)
Cu2—N11^vi^	2.044 (3)	C10—H10	0.9300
Cu2—N11	2.044 (3)	C11—C12	1.372 (5)
Cu2—N3	2.427 (3)	C11—C13	1.497 (6)
Cu2—N3^vi^	2.427 (3)	C12—H12	0.9300
Cu3—O1	1.943 (3)	C13—H13A	0.9600
Cu3—O1^vii^	1.943 (3)	C13—H13B	0.9600
Cu3—N10^vii^	2.015 (3)	C13—H13C	0.9600
Cu3—N10	2.015 (3)	C14—H14	0.9300
O1—H1	0.924 (19)	C15—C16	1.380 (7)
O1—H2	0.931 (19)	C15—H15	0.9300
O2—H3	0.950 (19)	C16—C17	1.385 (6)
O2—H4	0.933 (19)	C16—C18	1.508 (8)
N1—C1	1.146 (5)	C17—H17	0.9300
N1—Cu1^v^	1.962 (3)	C18—H18A	0.9600
N2—C2	1.115 (6)	C18—H18B	0.9600
N3—C3	1.145 (5)	C18—H18C	0.9600
			
C1—W1—C8	86.95 (13)	N10^vii^—Cu3—N10	179.999 (1)
C1—W1—C4	143.19 (14)	Cu3—O1—H1	129 (3)
C8—W1—C4	75.63 (14)	Cu3—O1—H2	122 (3)
C1—W1—C5	145.49 (14)	H1—O1—H2	106 (3)
C8—W1—C5	108.53 (15)	H3—O2—H4	102 (3)
C4—W1—C5	71.32 (15)	C1—N1—Cu1^v^	160.6 (3)
C1—W1—C3	96.29 (14)	C3—N3—Cu2	169.1 (3)
C8—W1—C3	145.84 (14)	C4—N4—Cu2^ii^	174.1 (3)
C4—W1—C3	81.95 (14)	C8—N8—Cu1	164.1 (3)
C5—W1—C3	87.73 (16)	C9—N9—C12	116.7 (3)
C1—W1—C7	80.17 (14)	C9—N9—Cu1	121.9 (3)
C8—W1—C7	69.75 (14)	C12—N9—Cu1	121.2 (3)
C4—W1—C7	121.55 (14)	C9—N10—C10	117.1 (3)
C5—W1—C7	76.96 (15)	C9—N10—Cu3	123.4 (2)
C3—W1—C7	144.35 (14)	C10—N10—Cu3	118.9 (2)
C1—W1—C6	73.46 (14)	C14—N11—C17	117.4 (4)
C8—W1—C6	140.10 (14)	C14—N11—Cu2	122.6 (3)
C4—W1—C6	138.18 (14)	C17—N11—Cu2	120.0 (3)
C5—W1—C6	75.27 (15)	C15—N12—C14	116.6 (4)
C3—W1—C6	72.22 (14)	N1—C1—W1	175.9 (3)
C7—W1—C6	72.80 (14)	N2—C2—W1	178.3 (5)
C1—W1—C2	71.57 (14)	N3—C3—W1	175.3 (3)
C8—W1—C2	75.22 (14)	N4—C4—W1	177.1 (4)
C4—W1—C2	72.72 (14)	N5—C5—W1	179.3 (4)
C5—W1—C2	141.37 (15)	N6—C6—W1	179.6 (4)
C3—W1—C2	73.68 (15)	N7—C7—W1	176.8 (4)
C7—W1—C2	135.68 (15)	N8—C8—W1	178.4 (3)
C6—W1—C2	127.18 (14)	N9—C9—N10	125.2 (3)
N1^i^—Cu1—N1^ii^	179.999 (1)	N9—C9—H9	117.4
N1^i^—Cu1—N9	93.24 (12)	N10—C9—H9	117.4
N1^ii^—Cu1—N9	86.76 (12)	N10—C10—C11	121.9 (3)
N1^i^—Cu1—N9^iii^	86.76 (12)	N10—C10—H10	119.1
N1^ii^—Cu1—N9^iii^	93.24 (12)	C11—C10—H10	119.1
N9—Cu1—N9^iii^	179.998 (1)	C12—C11—C10	116.3 (3)
N1^i^—Cu1—N8^iii^	90.31 (13)	C12—C11—C13	122.8 (4)
N1^ii^—Cu1—N8^iii^	89.69 (13)	C10—C11—C13	120.9 (4)
N9—Cu1—N8^iii^	89.69 (12)	N9—C12—C11	122.7 (3)
N9^iii^—Cu1—N8^iii^	90.31 (12)	N9—C12—H12	118.6
N1^i^—Cu1—N8	89.69 (13)	C11—C12—H12	118.6
N1^ii^—Cu1—N8	90.31 (13)	C11—C13—H13A	109.5
N9—Cu1—N8	90.31 (12)	C11—C13—H13B	109.5
N9^iii^—Cu1—N8	89.69 (12)	H13A—C13—H13B	109.5
N8^iii^—Cu1—N8	180.000 (1)	C11—C13—H13C	109.5
N4^iv^—Cu2—N4^v^	179.998 (1)	H13A—C13—H13C	109.5
N4^iv^—Cu2—N11^vi^	89.30 (13)	H13B—C13—H13C	109.5
N4^v^—Cu2—N11^vi^	90.70 (13)	N11—C14—N12	124.8 (5)
N4^iv^—Cu2—N11	90.70 (13)	N11—C14—H14	117.6
N4^v^—Cu2—N11	89.30 (13)	N12—C14—H14	117.6
N11^vi^—Cu2—N11	179.999 (1)	N12—C15—C16	123.5 (4)
N4^iv^—Cu2—N3	88.44 (13)	N12—C15—H15	118.3
N4^v^—Cu2—N3	91.56 (13)	C16—C15—H15	118.3
N11^vi^—Cu2—N3	90.62 (13)	C15—C16—C17	115.1 (4)
N11—Cu2—N3	89.38 (13)	C15—C16—C18	123.5 (5)
N4^iv^—Cu2—N3^vi^	91.56 (13)	C17—C16—C18	121.5 (5)
N4^v^—Cu2—N3^vi^	88.44 (13)	N11—C17—C16	122.6 (4)
N11^vi^—Cu2—N3^vi^	89.38 (13)	N11—C17—H17	118.7
N11—Cu2—N3^vi^	90.62 (13)	C16—C17—H17	118.7
N3—Cu2—N3^vi^	180.0	C16—C18—H18A	109.5
O1—Cu3—O1^vii^	180.00 (17)	C16—C18—H18B	109.5
O1—Cu3—N10^vii^	92.98 (12)	H18A—C18—H18B	109.5
O1^vii^—Cu3—N10^vii^	87.01 (12)	C16—C18—H18C	109.5
O1—Cu3—N10	87.02 (12)	H18A—C18—H18C	109.5
O1^vii^—Cu3—N10	92.98 (12)	H18B—C18—H18C	109.5
			
N4^iv^—Cu2—N3—C3	153.2 (18)	Cu2^ii^—N4—C4—W1	−113 (6)
N4^v^—Cu2—N3—C3	−26.8 (18)	C1—W1—C4—N4	24 (7)
N11^vi^—Cu2—N3—C3	63.9 (18)	C8—W1—C4—N4	−40 (7)
N11—Cu2—N3—C3	−116.1 (18)	C5—W1—C4—N4	−156 (7)
N3^vi^—Cu2—N3—C3	−35 (58)	C3—W1—C4—N4	114 (7)
N1^i^—Cu1—N8—C8	−24.6 (11)	C7—W1—C4—N4	−95 (7)
N1^ii^—Cu1—N8—C8	155.4 (11)	C6—W1—C4—N4	165 (7)
N9—Cu1—N8—C8	68.6 (11)	C2—W1—C4—N4	38 (7)
N9^iii^—Cu1—N8—C8	−111.4 (11)	C1—W1—C5—N5	13 (39)
N8^iii^—Cu1—N8—C8	−67 (100)	C8—W1—C5—N5	126 (39)
N1^i^—Cu1—N9—C9	79.9 (3)	C4—W1—C5—N5	−167 (100)
N1^ii^—Cu1—N9—C9	−100.1 (3)	C3—W1—C5—N5	−84 (39)
N9^iii^—Cu1—N9—C9	178 (28)	C7—W1—C5—N5	63 (39)
N8^iii^—Cu1—N9—C9	170.2 (3)	C6—W1—C5—N5	−12 (39)
N8—Cu1—N9—C9	−9.8 (3)	C2—W1—C5—N5	−144 (39)
N1^i^—Cu1—N9—C12	−104.5 (3)	C1—W1—C6—N6	−81 (62)
N1^ii^—Cu1—N9—C12	75.5 (3)	C8—W1—C6—N6	−17 (62)
N9^iii^—Cu1—N9—C12	−7 (28)	C4—W1—C6—N6	122 (62)
N8^iii^—Cu1—N9—C12	−14.2 (3)	C5—W1—C6—N6	85 (62)
N8—Cu1—N9—C12	165.8 (3)	C3—W1—C6—N6	177 (100)
O1—Cu3—N10—C9	−108.2 (3)	C7—W1—C6—N6	4 (62)
O1^vii^—Cu3—N10—C9	71.8 (3)	C2—W1—C6—N6	−131 (62)
N10^vii^—Cu3—N10—C9	56 (80)	C1—W1—C7—N7	−151 (6)
O1—Cu3—N10—C10	63.1 (3)	C8—W1—C7—N7	−60 (6)
O1^vii^—Cu3—N10—C10	−116.9 (3)	C4—W1—C7—N7	−3 (6)
N10^vii^—Cu3—N10—C10	−133 (80)	C5—W1—C7—N7	55 (6)
N4^iv^—Cu2—N11—C14	−55.8 (4)	C3—W1—C7—N7	122 (6)
N4^v^—Cu2—N11—C14	124.2 (4)	C6—W1—C7—N7	134 (6)
N11^vi^—Cu2—N11—C14	−19 (27)	C2—W1—C7—N7	−100 (6)
N3—Cu2—N11—C14	−144.2 (4)	Cu1—N8—C8—W1	−6 (14)
N3^vi^—Cu2—N11—C14	35.8 (4)	C1—W1—C8—N8	44 (13)
N4^iv^—Cu2—N11—C17	126.5 (3)	C4—W1—C8—N8	−168 (13)
N4^v^—Cu2—N11—C17	−53.5 (3)	C5—W1—C8—N8	−104 (13)
N11^vi^—Cu2—N11—C17	164 (27)	C3—W1—C8—N8	141 (12)
N3—Cu2—N11—C17	38.1 (3)	C7—W1—C8—N8	−36 (13)
N3^vi^—Cu2—N11—C17	−141.9 (3)	C6—W1—C8—N8	−15 (13)
Cu1^v^—N1—C1—W1	31 (5)	C2—W1—C8—N8	116 (13)
C8—W1—C1—N1	−173 (4)	C12—N9—C9—N10	−1.0 (6)
C4—W1—C1—N1	126 (4)	Cu1—N9—C9—N10	174.7 (3)
C5—W1—C1—N1	−54 (4)	C10—N10—C9—N9	1.0 (6)
C3—W1—C1—N1	41 (4)	Cu3—N10—C9—N9	172.4 (3)
C7—W1—C1—N1	−103 (4)	C9—N10—C10—C11	−0.3 (6)
C6—W1—C1—N1	−28 (4)	Cu3—N10—C10—C11	−172.1 (3)
C2—W1—C1—N1	112 (4)	N10—C10—C11—C12	−0.3 (6)
C1—W1—C2—N2	−17 (16)	N10—C10—C11—C13	178.8 (4)
C8—W1—C2—N2	−109 (16)	C9—N9—C12—C11	0.3 (6)
C4—W1—C2—N2	172 (16)	Cu1—N9—C12—C11	−175.4 (3)
C5—W1—C2—N2	150 (16)	C10—C11—C12—N9	0.3 (6)
C3—W1—C2—N2	85 (16)	C13—C11—C12—N9	−178.8 (4)
C7—W1—C2—N2	−70 (16)	C17—N11—C14—N12	−0.9 (7)
C6—W1—C2—N2	34 (16)	Cu2—N11—C14—N12	−178.6 (4)
Cu2—N3—C3—W1	17 (6)	C15—N12—C14—N11	0.2 (8)
C1—W1—C3—N3	−30 (4)	C14—N12—C15—C16	−0.1 (8)
C8—W1—C3—N3	−124 (4)	N12—C15—C16—C17	0.7 (8)
C4—W1—C3—N3	−173 (4)	N12—C15—C16—C18	−178.4 (6)
C5—W1—C3—N3	116 (4)	C14—N11—C17—C16	1.5 (7)
C7—W1—C3—N3	52 (5)	Cu2—N11—C17—C16	179.3 (4)
C6—W1—C3—N3	40 (4)	C15—C16—C17—N11	−1.4 (7)
C2—W1—C3—N3	−99 (4)	C18—C16—C17—N11	177.7 (5)

Symmetry codes: (i) −*x*+1, −*y*+2, −*z*+2; (ii) *x*+1, *y*, *z*; (iii) −*x*+2, −*y*+2, −*z*+2; (iv) −*x*+1, −*y*+2, −*z*+1; (v) *x*−1, *y*, *z*; (vi) −*x*, −*y*+2, −*z*+1; (vii) −*x*+2, −*y*+1, −*z*+2.

### Hydrogen-bond geometry (Å, º)

**Table:**

*D*—H···*A*	*D*—H	H···*A*	*D*···*A*	*D*—H···*A*
O1—H1···N6^viii^	0.92 (2)	1.86 (2)	2.771 (5)	167 (5)
O1—H2···O2	0.93 (2)	1.79 (2)	2.700 (4)	165 (4)
O2—H3···N12^ix^	0.95 (2)	2.00 (3)	2.914 (5)	161 (4)
O2—H4···N2^i^	0.93 (2)	2.02 (2)	2.944 (5)	169 (6)

Symmetry codes: (i) −*x*+1, −*y*+2, −*z*+2; (viii) −*x*+1, −*y*+1, −*z*+2; (ix) *x*+1, *y*, *z*+1.
